# Managing fatigue in inflammatory arthritis: a real-world evaluation of program length and delivery

**DOI:** 10.1093/rap/rkaf143

**Published:** 2025-12-03

**Authors:** Dervil M Dockrell, Kathryn Berg, Joanne Dobson, Helen Harris

**Affiliations:** Institute of Genetics and Cancer, Western General Hospital, Edinburgh, UK; Institute of Genetics and Cancer, Western General Hospital, Edinburgh, UK; Institute of Genetics and Cancer, Western General Hospital, Edinburgh, UK; Rheumatology Department, NHS Lothian, Western General Hospital, Edinburgh, UK

**Keywords:** fatigue management program, Scotland, sleep, mental health, online, outcome measures

## Abstract

**Objectives:**

Fatigue is a significant yet often overlooked symptom in rheumatic diseases, impacting sleep, mental health and daily functioning. The aim of this study was to evaluate the effectiveness of a fatigue management program (FMP) developed in NHS Lothian, comparing a 4-week format to a 7-week format. The study also examines the impact of in-person *vs* online delivery on patient outcomes.

**Methods:**

A retrospective analysis was conducted on 22 FMPs delivered between February 2020 and March 2024. Patients with inflammatory arthritis were referred to the program, with 142 patients participating. Initially, sessions were in-person, but due to COVID-19, the program transitioned online and was shortened from 7 to 4 weeks in response to high demand. Standardised patient-reported outcome measures (PROMs) assessed fatigue severity, fatigue impact, mental health and sleep quality. A control group (*n* = 16) received usual care (fatigue booklet).

**Results:**

Both the 4-week and 7-week FMPs significantly improved fatigue severity (*P* < 0.01), fatigue impact (*P* < 0.01), mental health (*P* < 0.01) and sleep quality (*P* < 0.01). No significant differences were found between the 4-week and 7-week programs (*P* > 0.05), nor between online and in-person delivery (*P* > 0.05). The control group showed no significant improvement in any measure.

**Conclusion:**

A 4-week FMP is as effective as a 7-week program, making it a more accessible option. Virtual delivery provides an equally beneficial alternative to in-person sessions. These findings support the use of shorter, remotely delivered FMPs as a scalable, resource-efficient approach to fatigue management in rheumatology.

Key messages

- A fatigue management program (FMP) improves fatigue, mental health and sleep quality in rheumatology patients.
- A 4-week FMP is equivalent to a 7-week FMP and better than usual care.
- There are no significant differences in patient improvement outcomes between online and in-person FMPs.

## Introduction

Fatigue is a significant problem for patients with rheumatic diseases (RDs) but remains overlooked and undertreated in real-world settings [1–3]. Fatigue is known to be associated with increased sleep disturbance and poorer mental health in RDs [4, 5]. Despite its prevalence, access to treatment varies across the UK [6], hindered by a lack of healthcare professional (HCP) knowledge [7] and limited staffing resources within rheumatology departments [8]. No guidelines exist for managing fatigue in RDs.

The Rheumatoid Arthritis Fatigue Trial (RAFT) compared a group-based cognitive behavioural approach (CBA) and usual care with usual care alone in RA and found that the CBA group was more effective at managing fatigue than the usual care group [9]. The Lessening the Impact of Fatigue (LIFT) Study examined a one-on-one CBA or a personal exercise program by telephone, finding that CBA significantly reduced fatigue severity and impact [10]. An evaluation of the Fatigue and Activity Management Education program (*n* = 21) supported group programs for SLE, reporting reduced depression and improved quality of life [11]. Previous research also suggests virtual fatigue management programs (FMPs) improve fatigue [12].

The NHS Lothian (NHSL) FMP was developed as part of Lupus UK funding in 2019. It was adapted over 3 years in response to patient feedback and increasing demand. The COVID-19 pandemic required healthcare professionals (HCPs) to find alternative solutions to in-person appointments. Based on the RAFT study [9], the NHSL FMP involved in-person groups meeting for 2-h each week over 6 weeks, with a review 2 months after completion. The FMPs were delivered by a rheumatology nurse and occupational therapist (OT) who completed training in cognitive behavioural therapy (CBT), CBT for insomnia (CBTi) and the RAFT training course.

The FMP followed weekly topics: (1) fatigue and you, (2) sleep and the REST™ program, (3) thoughts, emotions and behaviours, (4) activity and the 4P’s (prioritise, plan, pace, posture), (5) Stress and communication, (6) coping with setbacks and (7) progress review and feedback. Sessions included education, cognitive exercises and group discussions. Patients set weekly goals and shared their experiences.

In May 2020, the NHSL FMP was moved online due to COVID-19 restrictions. Due to an increased number of referrals, the program was shortened to 4 weeks to increase capacity and reduce patient time commitment. Some FMPs were held virtually.

The purpose of this study is to describe the adaptations made in response to feedback, to report the results of the service evaluation conducted, to examine whether a 4-week FMP is as effective as a 7-week FMP and to evaluate the impact of the FMP on patient reported outcome measures (PROMs).

## Methods

### Study design

This report provides a retrospective analysis of 22 FMPs that were delivered in the NHSL between February 2020 and March 2024. Data collection was approved by East of Scotland Research Ethics Service REC 2 (24/ES/0084). Patients consented to taking part in the FMP and all data were anonymised prior to analysis for the purpose of this evaluation.

### Participants

Patients from the NHSL rheumatology clinics with a diagnosis of an inflammatory arthritis and a visual analogue scale (fatigue) (VAS-F) score of ≥6 could be referred into the FMP.

### Data collection

Standardised PROMs were used to measure fatigue, mental health, sleep, self-efficacy and quality of life. These were completed at baseline and at the end of the program and returned by post. PROMs were adjusted in 2021 in response to a service evaluation to capture the impact of fatigue rather than simply its severity. Anonymous patient feedback questionnaires were returned at the end of each FMP. Patients who could not attend the group program for various reasons were offered usual care and were utilised as controls for the purpose of this study.

### Data analysis

Paired *t*-tests were used to determine any difference between the PROM results reported at baseline and at the end of the FMPs. Unpaired *t*-tests were used to compare between different formats. All data analysis was completed using Minitab Statistical Software 22 (Minitab, State College, PA, USA).

## Results

### Demographics and clinical characteristics

During the study period, 378 referrals were received from the rheumatology team at NHSL for patients diagnosed with an inflammatory arthritis. Of these, 142 patients were allocated to an FMP following the return of baseline questionnaires. A total of 125 patients were unable to attend the FMP and were referred to an occupational therapist for individual support. A smaller standard-care arm (*n* = 16), used as an exploratory comparator, was comprised of patients who were unable to participate in an FMP and were directed to the Versus Arthritis fatigue booklet, which is considered usual care in the NHSL. The remaining patients either declined treatment or did not respond to the invitation. The vast majority (84.5%) of patients allocated to an FMP were female and the average age was 50.62 years (s.d. 13.88). The inflammatory arthritis most represented in this sample was RA, with 51 patients having this diagnosis. Other diagnoses were SLE (*n* = 38), PsA (*n* = 14), primary Sjögren’s disease (*n* = 10) and axial SpA (*n* = 10). Other diagnoses reflected in this sample were Bechet’s, scleroderma, SSc, JIA, Takayasu arteritis, PMR and MCTD.

### Impact of the NHSL FMP on fatigue and impact of fatigue (Table 1)

Both the 4-week and 7-week FMP groups completed the Fatigue Severity Scale (FSS) and VAS-F at each time point. There were significant improvements in fatigue severity measured by the FSS (*P =*  <0.01) and VAS-F (*P* =<0.01) in both groups. In 2021, it was determined that there were no significant differences between the 4-week and 7-week FMPs in terms of improvement in fatigue severity and all programs were moved to a 4-week format to accommodate lengthening waiting lists. The impact of fatigue on physical, cognitive and psychosocial functioning was measured in subsequent 4-week groups using the Modified Fatigue Impact Score (MFIS). The Fatigue Impact Visual Analogue Score (FI-VAS) was used to measure the impact of fatigue on quality of life. There were significant improvements in both the MFIS (*P* =<0.01) and FI-VAS (*P* = <0.01) between baseline and review.

**Table 1:** PROMs at baseline and review in each group.

PROMs	4-week baseline	4-week review	*P*-value	7-week baseline	7-week review	*P*-value	Usual care baseline	Usual care review	*P*-value
FSS	51.45 ± 1.97	41.95 ± 2.40	<0.01	53.36 ± 1.50	46.16 ± 1.82	<0.01			
VAS-F	3.31 ± 0.39	5.36 ± 0.37	<0.01	3.00 ± 0.37	5.55 ± 0.37	<0.01			
PHQ9	12.78 ± 1.40	8.39 ± 1.23	<0.01	14.48 ± 1.29	9.36 ± 1.12	<0.01			
GAD-7	8.63 ± 1.47	6.00 ± 1.36	0.038	8.80 ± 1.35	5.68 ± 1.02	<0.01			
PSQI	11.14 ± 0.56	8.75 ± 0.52	<0.01	11.60 ± 1.01	9.24 ± 0.89	<0.01			
MFIS^a^	57.65 ± 1.90	46.83 ± 2.25	<0.01				55.13 ± 4.24	53.81 ± 4.69	0.56
FI-VAS^a^	8.13 ± 0.20	6.85 ± 0.29	<0.01				7.31 ± 0.46	7.31 ± 0.48	1.00
PHQ4^a^	5.08 ± 0.40	3.34 ± 0.37	<0.01						

Values are reported as mean ± SEM.

a These PROMs were introduced in 2021 in response to a service evaluation and patient feedback.

### Impact of the NHSL FMP on mental health and sleep quality (Table 1)

The 9-item Patient Health Questionnaire (PHQ9) was utilised as a means of measuring depression in the study population. Early 7-week and 4-week groups reported a significant improvement in PHQ9 scores at review (*P=*  <0.01). The 7-item Generalized Anxiety Disorder (GAD-7) tool was used to assess levels of anxiety in the study population. There were significant improvements in both the 4-week group (*P* = 0.038) and the 7-week group (*P=* < 0.01) at the review time point. Patient feedback indicated the questionnaire burden was too high, therefore later groups were asked to complete a 4-item Patient Health Questionnaire (PHQ4) that measures both anxiety and depression in place of the PHQ9 and GAD-7. There was a significant improvement in scores at the review (*P=* <0.01) using the PHQ4. Sleep quality was measured in both groups using the Pittsburgh Sleep Quality Index (PSQI) and there was significant improvement (*P* = <0.01) following the FMPs. A previous service evaluation found a significant correlation between the reduction in fatigue, as measured by VAS-F scores, and improvement in sleep quality as measured by the PSQI (*r* = 0.365) [13].

**Figure 1:**
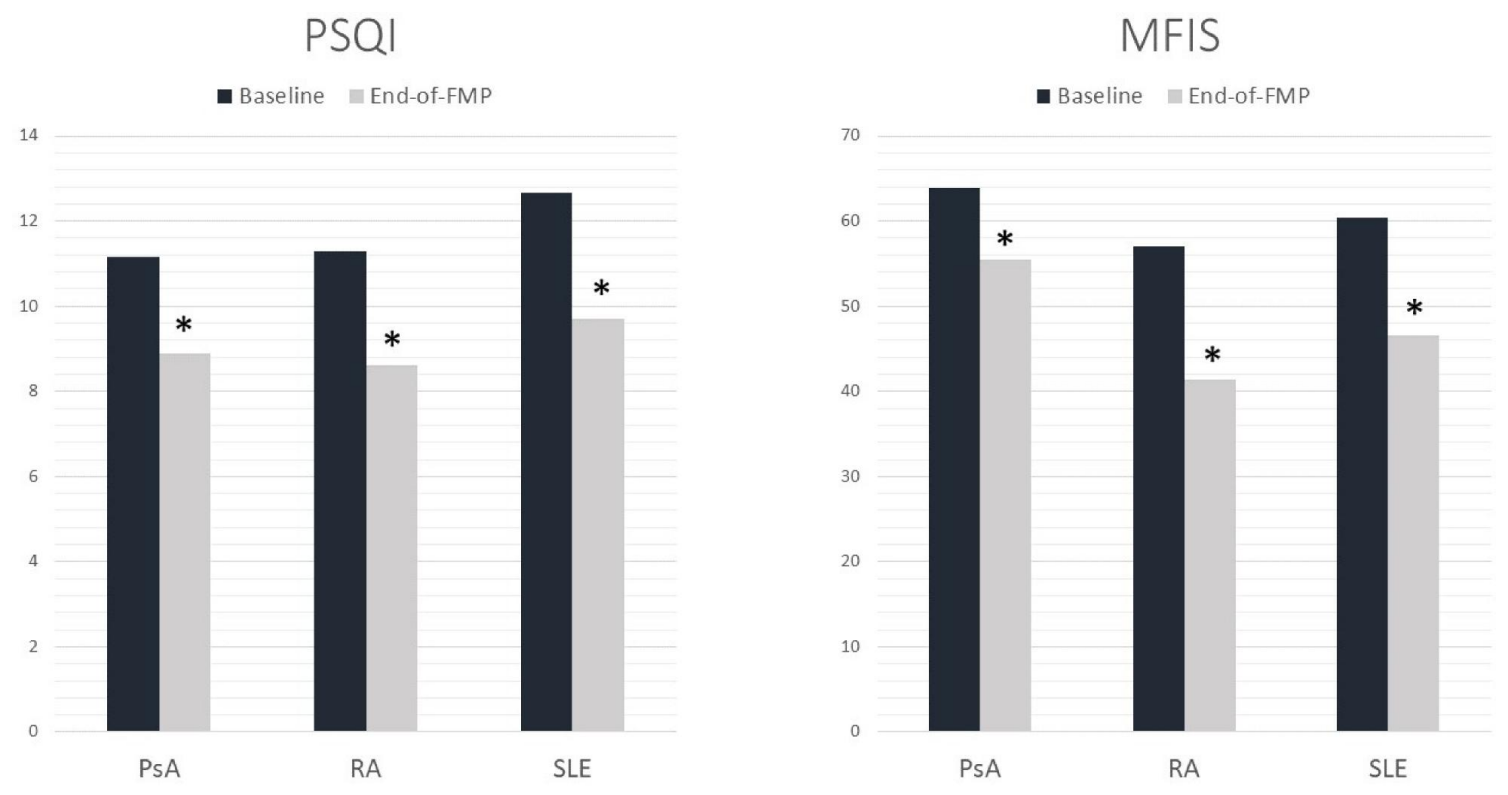
Mean PROM scores in the three most-represented disease groups. *P =<0.05

A subgroup analysis found that results were consistent across the three most represented disease groups—RA, SLE and PsA (see Fig. 1)—which significantly improved in sleep- and fatigue-impact PROMs (*P=<* 0.05).

### Comparing the 4-week FMP to the 7-week FMP

There were no significant differences in patient improvement between the 4-week FMP and the 7-week FMP in any of the comparable measures: FSS (*P* = 0.393), VAS-F (*P* = 0.468), PHQ9 (*P* = 0.748), GAD-7 (*P* = 0.682) and PSQI (*P* = 0.963) (Supplementary Table S1, available at *Rheumatology Advances in Practice* online).

### Comparing the 4-week FMP to usual care

Patients allocated to usual care completed the MFIS and FI-VAS at baseline and 8 weeks. These patients reported no significant improvement in either MFIS (*P* = 0.56) or FI-VAS (*P* = 1.00). The differences in improvement between groups were assessed using an unpaired *t*-test. Patients in the 4-week group reported a significantly greater improvement in both the MFIS (*P=* < 0.01) and the FI-VAS (*P* = 0.01) when compared with usual care (Supplementary Table S1, available at *Rheumatology Advances in Practice* online).

### Comparing a live-online FMP format to a face-to-face FMP format

There were no significant differences in patient improvement between the patients who attended an FMP online and the patients who attended an in-person FMP at any of the comparable measures: FSS (*P* = 0.217), VAS-F (*P* = 0.418), PHQ9 (*P* = 0.247), GAD-7 (*P* = 0.391) and PSQI (*P* = 0.974) (Supplementary Table S1, available at *Rheumatology Advances in Practice* online).

## Discussion

This work demonstrates the significant impact of providing a fatigue management program as a treatment option to patients living with inflammatory RDs when compared with usual care. FMPs evidence improvement beyond fatigue, equipping patients with valuable strategies that enhance many aspects of their lives. The NHSL FMP was responsive, adapting to changes in healthcare delivery triggered by the COVID-19 pandemic, allowing a unique opportunity to compare and evaluate a number of different formats.

Previous studies into fatigue management interventions utilised a 6-week FMP (RAFT) and an 8-week FMP (LIFT) [10]. A key finding of our study was that a reduced program consisting of 3 weeks of education followed by review is just as effective at lessening the impact of fatigue as an extended program. The most common approach to self-management education within the NHS consists of 6–8 weeks of patient education with limited opportunity for follow-up [14]. In the NHSL, condensing the program allowed us to dramatically reduce waiting lists, increase staff capacity and reduce patient burden and time commitment. There were also no significant differences found between patients who attended an in-person FMP and a live-online FMP, further evidencing that virtual treatment is effective. The importance of this finding lies in the fact that FMPs could be delivered to patients with minimal resources, freeing up rooms, increasing retention and reducing the additional burden of attending appointments.

The EULAR recommendations for fatigue management in RDs include tailored psychoeducational interventions and advise that HCPs also consider patients’ needs and preferences [15]. In December 2023, we sent an online survey to RD patients (*n* = 45), asking them if they would have liked a choice in the fatigue management option they were given. Of those who responded, 80% indicated that they would like the opportunity to choose which intervention they would prefer. The most popular choice among those respondents was the live-online group fatigue management program, with 63% indicating this as their first choice and 9% indicating this as their second choice [16].

Although this retrospective analysis has offered the opportunity to compare multiple FMP formats, there are some limitations. Comparisons with the usual care group should be interpreted cautiously given its small sample size and exploratory nature. Although within expected response rate ranges for postal surveys [17], there was varying return of PROMs following the programs. The face-to-face format offered better follow-up PROM completion rates (93%) compared with the live-online group (60%). This evaluation focusses on short-term outcomes, and future research should consider long-term outcomes to assess the effectiveness of FMPs as a lifelong management tool in RDs. The ongoing Fatigue in Lupus Intervention Programmes study [18] will evaluate PROMs from identical FMPs in SLE patients up to 12 months post-intervention.

The NHSL FMP has demonstrated significant benefits for patients with inflammatory RDs, reducing fatigue severity, improving mental health and enhancing sleep quality. Crucially, this study found that a condensed 4-week FMP was just as effective as the traditional format, enabling greater accessibility while maintaining efficacy. A live-online group setting could be achieved within the remit of most rheumatology occupational therapy departments and was preferred by most patients seeking fatigue management in the NHSL. The findings support the potential scalability of short, virtual FMPs across other NHS boards and international healthcare systems and underline the importance of incorporating patient preference into service design.

## Supplementary Material

rkaf143_Supplementary_Data

## Data Availability

The data underlying this article will be shared upon reasonable request to the corresponding author.
