# Supplementary material for: Managing fatigue in inflammatory arthritis: a real-world evaluation of program length and delivery
Source: Rheumatol Adv Pract. 2025 Dec 3;10(1):rkaf143. doi: 10.1093/rap/rkaf143 (PMC12758122; doi:10.1093/rap/rkaf143)
Supplement: rkaf143_Supplementary_Data [file rkaf143_supplementary_data.docx]

|  | 4-Week | 7-Week | p-value | Usual Care | p-value* | Virtual | F2F | p-value |
| --- | --- | --- | --- | --- | --- | --- | --- | --- |
| *Fatigue Severity Scale (FSS)* | -9.50 ± 1.99 | -7.20 ± 1.78 | 0.393 |  |  | -8.63 ± 1.57 | -5.00 ± 20.9 | 0.217 |
| *Visual Analogue Scale (Fatigue) (VAS-F)* | 2.06 ± 0.47 | 2.55 ± 0.46 | 0.468 |  |  | 2.20 ± 0.34 | 2.80 ± 0.80 | 0.418 |
| *Patient Health Questionnaire (PHQ9)* | -4.63 ± 1.26 | -5.12 ± 0.91 | 0.748 |  |  | -4.07 ± 0.68 | -5.91 ± 1.81 | 0.247 |
| *Generalised Anxiety Disorder Assessment (GAD7)* | -2.50 ± 1.13 | -3.12 ± 1.00 | 0.682 |  |  | -2.33 ± 0.90 | -3.82 ± 1.39 | 0.391 |
| *Pittsburgh Sleep Quality Index (PSQI)* | -2.40 ± 0.44 | -2.36 ± 0.63 | 0.963 |  |  | -2.15 ± 0.37 | -2.18 ± 0.78 | 0.974 |
| *Modified Fatigue Impact Score (MFIS) ^* | -10.82 ± 1.64 |  |  | -1.31 ± 2.22 | <0.01 |  |  |  |
| *Fatigue Impact Visual Analogue Score (FI-VAS) ^* | -1.28 ± 0.26 |  |  | 0.00 ± 0.24 | 0.01 |  |  |  |

**Supplementary Table S1. Comparison between changes in PROMs at different FMP formats: 4-week vs. 7-week, *4-week vs. Usual Care, and Virtual vs. Online. Figures are reported as Mean ± SEM.**^ These PROMs were introduced in 2021 in response to a service evaluation and patient feedback
